# Relationship of Serum Uric Acid Level and Angiographic Severity of Coronary Artery Disease in Male Patients with Acute Coronary Syndrome

**DOI:** 10.12669/pjms.295.4029

**Published:** 2013

**Authors:** Azmat Ehsan Qureshi, Shahid Hameed, Ahmed Noeman

**Affiliations:** 1 Azmat Ehsan Qureshi, MBBS, FCPS (MED), Post graduate trainee for FCPS Cardiology, Punjab Institute of Cardiology, Lahore, Pakistan.; 2 Shahid Hameed, MBBS, MRCP (UK), Associate Professor of Cardiology, Punjab Institute of Cardiology, Lahore, Pakistan.; 3 Ahmed Noeman, MBBS, FCPS (MED), FCPS(Cardiology), Assistant Professor of Cardiology, Punjab Institute of Cardiology, Lahore, Pakistan.

**Keywords:** Acute coronary syndrome, CVD risk factors, Gensini score, Serum uric acid

## Abstract

***Background and objective:*** The association between serum uric acid and ischemic heart disease remains controversial and it has not yet been established as cardiovascular risk factor. Our objective was to study the association of serum uric acid level with angiographic severity of coronary artery disease in men with acute coronary syndrome (ACS).

***Methods*** **:** This cross-sectional study was conducted on 100 consecutive male patients presenting with ACS at Punjab Institute of Cardiology. Hyperuricemia was defined as serum uric acid level > 6.5 mg/dl. Severity of ischemic heart disease was assessed on the basis of Gensini score, number of diseased vessels, critical lesions and total occlusions on coronary angiogram.

***Results: ***Mean age of normouricemic group (n=59) was 52.62 ± 9.46 years and mean age of hyperuricemic group (n=41) was 50.52 ± 9.40 years (p=0.273). Mean uric acid level; normouricemic group (4.75 ± 1.05), hyperuricemic group (7.61 ± 1.24), p<0.001. Mean Gensini score; normouricemic group (22.15 ± 21.52), hyperuricemic group (35.69 ± 26.80). Mann Whitney U test was applied to compare the Gensini score of two groups and it showed statistically significant difference (p value <0.006). Critical lesions, total occlusions and multi-vessel disease were more frequent in hyperuricemic group but statistically significant difference was found only for total occlusions (p=0.013) and critical lesions (p=0.046).

***Conclusions*** **: **Hyeruricemia is associated with higher Gensini score and more frequent total occlusions and critical lesions in men presenting with acute coronary syndrome.

## INTRODUCTION

An association between serum uric acid and cardiovascular disease (CVD) has been observed since 19th century. Since then huge epidemiologic data showing relation of uric acid with variety of CVDs has accumulated. It has also been observed that hyperuricemia is associated with cardiovascular risk factors for ischemic heart disease like male sex, old age, diabetes mellitus, hypertension, insulin resistance, hypertriglyceridemia and metabolic syndrome.^1^ It has also been proved in different studies that there is strong association between serum uric acid level and adverse outcomes in ischemic heart disease (IHD) especially in patients with heart failure.^2^

Uric acid is the end product of purine metabolism. Its immediate precursor, xanthine, is converted to uric acid by an enzymatic reaction involving xanthine oxidoreductase.^3^ It has been found that increased serum uric acid is associated with endothelial dysfunction,^4^ anti- proliferative effects, high oxidative stress, generation of free radicals^5^ and thrombus formation,^6^ all promoting atherosclerosis and its sequelae. Endothelial dysfunction is regarded as the main mechanism by which hyperuricemia promotes atherosclerosis. Patients with persistently elevated levels of uric acid in blood serum have significantly higher levels of endothelial dysfunction (ED) markers; albuminuria and plasma endothelin.^7^

Despite this evidence, uric acid has yet not been recognized as a CVD risk factor by major professional societies, and treatment of asymptomatic hyperuricemia to reduce CVD risk is not advised.

The objectives of our study were to study the association of serum uric acid level with IHD in local population and study its relationship with severity of coronary artery disease (CAD).

## METHODS

This was a cross-sectional comparative study of 100 consecutive male patients (Age 30-70 yrs) presenting with ACS at Punjab institute of cardiology Lahore between February 2012 and May 2012. Written informed consent was taken and study was approved by ethical review committee. Demographic, clinical, procedural, and laboratory data were collected. CVD risk factors were recorded according to standard definitions. ACS patients were; unstable angina, NSTEMI and STEMI.

Exclusion criteria included a history of IHD, heart failure, liver and kidney diseases, hematological or oncological disorders and chronic infections. Patients taking diuretics, multivitamins, alcohol and on drugs interfering with serum uric acid levels were also excluded.

Serum uric acid level was done after >8 hrs of fasting and analysis was by photometric technique with normal reference range of 3.5-6.5 mg/dl. Coronary angiography was performed during same hospital admission.

***Coronary angiography: ***Coronary angiography was performed through right femoral route by using Judkin’s technique and on Bio-core and HI-core (Siemens, Germany) and Integrus (Philips, Netherlands) machines in catheterization laboratory of Punjab Institute of Cardiology Lahore. It was done by two experienced cardiologists. Reporting of coronary angiogram was done by a consultant cardiologist not aware of clinical or biochemical profile of patients. The Gensini score was used to evaluate the severity of atherosclerotic lesions on angiogram. The Gensini score was computed by assigning a severity score to each coronary stenosis according to the degree of luminal narrowing (visual assessment) (Table-I).

**Table-I:** Gensini number assigned according to degree of luminal narrowing

*Luminal Narrowing (%)*	*Grading of disease*	*Gensini number*
30-50	Mild	01
51-70	Moderate	02
71-90	Severe	04
91-99	Subtotal occlusion	08
100	Total occlusion	16

This number is then multiplied by a factor that takes into account the geographical importance of the lesion position in the coronary arterial tree (Table-II).

**Table-II:** Gensini multiplying factor assigned according to the location of lesion in the coronary tree

*Location of lesion*	*Multiplying factor*
Left main stem	05
Proximal LAD and proximal LCX	2.5
Mid LAD	1.5
Distal LAD, first diagonal, mid LCX, distal LCX, obtuse marginal, proximal RCA, mid RCA, distal RCA,PDA	1.0
Second diagonal, PLV	0.5

LAD: Left anterior descending artery. LCX: Left Circumflex artery.

RCA: Right coronary artery. PDA: Posterior descending artery.

PLV: Posterolateral ventricular branch.

**Table-III:** Comparison between Normouricemic and Hyperuricemic groups

*Parameter*	*Normouricemic(n=59)*	*Hyperuricemic(n=41)*	*P value*
Mean uric acid level	4.71±1.05	7.61±1.24	*<0.001*
Age	52.62± 9.46	50.52±9.40	*0.273*
Diabetics	18(30.5%)	15(36.5%)	*0.525*
Hypertensives	18(30.5%)	18(44.9%)	*0.170*
Smokers	13(22.03%)	09(21.9%)	*0.992*
+ve family history	07(11.8%)	03(7.3%)	*0.456*
Hyperlipidemics	06(10.1%)	03(7.3%)	*0.624*
Unstable angina	01(1.6%)	03(7.3%)	*0.158*
NSTEMI	31(52.5%)	19(46.3%)	*0.542*
STEMI	27(45.7%)	19(46.3%)	*0.954*
Gensini score	22.15 ± 21.52	35.69 ± 26.80	*0.006*
Diseased vessels	2.03 ± 0.95	2.21±0.91	*0.329*
Critical lesions	0.66 ± 0.82	1.04 ± 1.09	*0.046*
Total occlusions	0.32 ±0.60	0.68 ± 0.82	*0.013*

**Table-IV:** Comparison of Serum Uric Acid Levels in Patients with Established Risk Factors

*Serum uric acid level*		*P value*
*Diabetic Non-diabetic*		
6.15 ± 1.79	5.71 ± 1.81	0.312
*Hypertensive Non-hypertensive*		
5.74 ±2.04	5.94 ± 1.72	0.608
*Smoker Non-smoker*		
5.68 ±1.42	5.96 ± 1.90	0.515
*+ F/H - F/H*		
6.07 ± 2.19	5.88 ± 1.77	0.763
*Hyperlipidemic Non-hyperlipidemic*		
5.30 ± 2.37	5.96 ± 1.74	0.295

**Fig.1:**
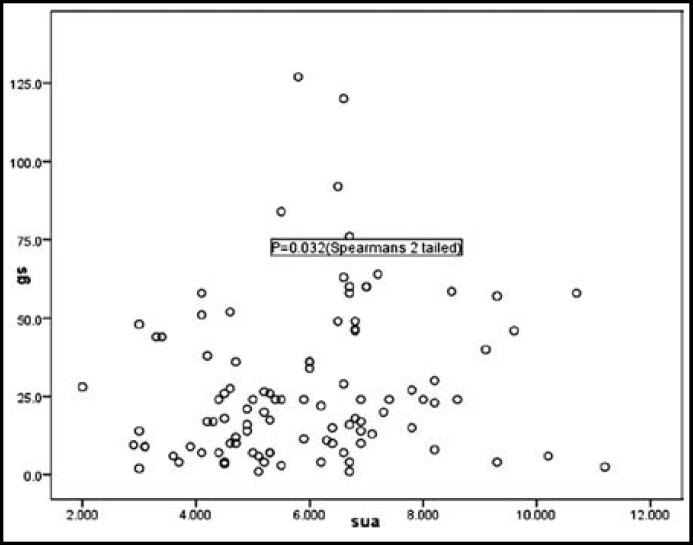
Correlation between Serum uric acid levels (mg/dl) and Gensini score (p=0.032).

The Gensini score was then expressed as sum of scores of all coronary arteries. Critical lesion was defined as ≥70% stenosis involving proximal part of any of the three main coronary arteries or ≥50% of left main stem stenosis. Total occlusion was defined as 100% occlusion with no ante grade flow of contrast distal to the lesion. A vessel was counted as diseased vessel if it has ≥50% stenosis at any level.

***Statistical Analysis: ***The analysis was performed using SPSS V 16.0 for windows. Quantitative data was expressed as mean value ± 1 standard deviation. Qualitative variables were presented by calculating frequency and percentage. Data was stratified for DM, HTN, Smoking, family history to address effect modifiers. Comparison of Gensini score between two groups was performed by Mann–Whitney U test. A 2-tailed P value less than 0.05 was considered statistically significant.

## RESULTS

Among 100 male patients; 59 (59%) were normouricemic (serum uric acid <6.5 mg/dl) and 41 (41%) were hyperuricemic (serum uric acid > 6.5mg/dl) (Table-III). Mean age; normouricemic group (52.62 ± 9.46 years), hyperuricemic group (50.52 ± 9.40 years), p value 0.273.

Mean uric acid levels of normouricemic and hyperuricemic groups were 4.75 ± 1.05 and 7.61 ± 1.24 respectively (Table-III). Mean Gensini score; normouricemic group (22.15 ± 21.52), hyperuricemic group (35.69 ± 26.80). Mann Whitney U test was applied to compare the Gensini score of 2 groups and it resulted in p valve of 0.006 which shows statistically significant difference between 2 groups. Direct correlation between serum uric acid levels and Gensini score was also found to be statistically significant using 2-tailed Spearman correlation (Fig.1). Hyperuricemia was more frequently but not statistically significantly associated with multivessel disease. However there was significant difference regarding total occlusions (P=0.013) and critical lesions (P=0.046).

Frequency of established cardiovascular risk factors in both groups was comparable (Table-III). Eighteen (30.5%) patients in normouricemic group were diabetic as compared to 15 (36.5%) in hyperuricemic group (P= 0.52) while 18 (30.5%) were hypertensive in normouricemic group as compared to 18 (44.9%) in hyperuricemic group (P=0.17). Serum uric acid levels were comparable across the whole spectrum of study population; with or without established CVD risk factors (Table-IV).

## DISCUSSION

In this observational study, hyperuricemic men had higher Gensini score, showing association of uric acid with severity of CAD. We documented CAD severity by coronary angiography which is the gold standard for diagnosis of IHD.

In the present study Gensini scoring system has been used for assessing angiographic severity of IHD. The Gensini scoring system and the Syntax scoring system are the two most widely used scoring systems for assessing angiographic severity and complexity of IHD.^8^ Studies have proved their correlation and comparability, with none of them inferior to the other.^9^ Gensini scoring system which, first introduced in 1975 gives weightage to the proximity of lesions within the coronary tree in addition to the degree of stenosis, the lesions in left main coronary artery getting the maximum score.^10^ The final score is a true reflection of severity of ischemic heart disease as it sums up cumulative effect of all lesions. It has also been shown to give more valuable prognostic information.

In a recent study in Turkey, Deveci et al^11^ evaluated 1012 patients for association of serum uric acid with angiographic presence and severity of IHD using Gensini score. A statistically significant difference in the mean uric acid concentrations was found between the patients with and without coronary artery disease irrespective of sex. Also there was significant association of severity of IHD with serum uric acid levels. In a similar study also from Turkey a series of 495 patients of stable IHD were studied by Gur M et al^12^ for an association between hyperuricemia and angiographic severity of IHD using Gensini score. They observed positive correlation between serum uric acid levels and angiographic presence of IHD but not with severity of ischemic heart disease. Duran M et al^13^ studied 246 non-diabetic and non-hypertensive patients of ACS and found a positive association of hyperuricemia with angiographic severity of ischemic heart disease (Gensini score). Results obtained in these studies are comparable with our data. Xiong Z et al^14^ found hyperuricemia to be prominently related to angiographic complexity (not severity) of coronary artery disease (Syntax score). Contrary to our results, from China Lu P et al^15^ reported that there was no relationship between serum uric acid levels and severity of IHD. This study was done in stable IHD patients.

Increased serum uric acid levels have been observed in diabetics, hypertensive, obese and dyslipidemics. A multiple risk factor clustering syndrome has been considered to be responsible for high serum uric acid level in IHD questioning the role of serum uric acid level as independent risk factor.^16^ Most studies in the past have excluded diabetics and hypertensives for the same reason.^15^ In our study there was no statistically no difference in serum uric acid levels of diabetics vs. non-diabetics and hypertensives vs. non-hypertensives suggesting the role of serum uric acid as an independent factor for severity of IHD in patients with ACS.

Hyperuricemia has also been studied as a marker of poor prognosis and increased mortality in general population,^17^ other co-morbidities and established IHD patients. In a recent study by Ndrepepa G et al^18^, of over 5000 patients with ACS undergoing PCI, raised serum uric acid was associated with increased 1 year mortality; with 12 % increase in the adjusted risk for every 1 mg/dl increase in serum uric acid level. And this increased risk was observed in all ACS groups (STEMI, NSTEMI and USA). Kojima S et al^19^ also showed that serum uric acid predicted the development of heart failure and long term mortality in acute MI. This risk was even stronger in women.^19^

Uric acid in ACS has also been shown to predict ICU complications, mortality in pre-existing renal disease and contrast induced nephropathy in patients undergoing invasive procedures. Other than ischemic heart disease serum uric acid level has also been studied for association with other cardiovascular diseases like atrial fibrillation, dilated cardiomyopathy, calcific aortic stenosis and metabolic syndrome and results are convincing for these associations.^19^^,^^20^

Recent data has shown renewed interest in the inhibition of xanthine oxidase system for preventing progression of atherosclerosis.^21^ Allopurinol a xanthine oxidase inhibitor is being extensively studied for its role as add-on therapy in stable IHD.^22^ It acts by reducing vascular oxidative stress resulting in endothelial stabilization. Studies have also proven its beneficial effects in prolonging survival in patients with heart failure.^23^ Use of allopurinol is also associated with reduction in LV mass, thereby indirectly relieving angina symptoms.^24^ Rajendra NS et al^25^ observed reduction in oxidative stress with allopurinol at tissue level, but its translation into clinical benefit needs further data. Beneficiaries of allopurinol are not only the coronary arteries but the whole cardiovascular system; reduction in ischemic strokes with use of allopurinol has also been reported.^26^

In conclusion, this study has shown association of severity of CAD with hyperuricemia. The limitations of the study were only male patients, serum uric acid level was done once, study population was a limited number, and it included only patients with ACS. It would also be interesting to follow up patients for prognostic significance, and study the effect of varying uric acid levels.

## References

[1] Rathmann W, Funkhouser E, Dyer AR, Sesti G, Perticone F, Pujia A (1998). Relations of hyperuricemia with the various components of the insulin resistance syndrome in young black and white adults: the CARDIA study. Ann Epidemiol.

[2] Hamaguchi S, Furumoto T, Tsuchihashi-Makaya M, Goto K, Goto D, Yokota T, JCARE-CARD Investigators (2011). Hyperuricemia predicts adverse outcomes in patients with heart failure. Int J Cardiol.

[3] Gertler MM, Garn SM, Levine SA (1951). Serum uric acid in relation to age and physique in health and in coronary heart disease. Ann Intern Med.

[4] Diaz MN, Frei B, Vita JA, Keaney JF (1997). Antioxidants and atherosclerosis heart disease. N Engl J Med.

[5] Anker SD, Leyva F, Poole-Wilson PA, Kox WJ, Stevenson JC, Coats AJ (1997). Relation between serum uric acid and lower limb blood flow in patients with chronic heart failure. Heart.

[6] Kim SY, Guevara JP, Kim KM, Choi HK, Heitjan DF, Albert DA (2010). Hyperuricemia and coronary heart disease: a systematic review and meta-analysis. Arthritis Care Res.

[7] Clinical implication of endothelial dysfunction in patients with essential arterial hypertension and urate dysbolism with renal damage (2011). Ter Arkh.

[8] Neeland IJ, Patel RS, Eshtehardi P, Dhawan S, McDaniel MC, Rab ST (2012). Coronary Angiographic Scoring Systems-An Evaluation of Their Equivalence and Validity. Heart J.

[9] Sinning C, Lillpopp L, Appelbaum S, Ojeda F, Zeller T, Schnabel R (2013). Angiographic score assessment improves cardiovascular risk prediction: the clinical value of SYNTAX and Gensini application. Clin Res Cardiol.

[10] Masood A, Jafar SS, Akram Z (2011). Serum high sensitivity C-reactive protein levels and the severity of coronary atherosclerosis assessed by angiographic gensini score. J Pak Med Assoc.

[11] Deveci OS, Kabakci G, Okutucu S, Tulumen E, Aksoy H, Kaya EB (2010). The association between serum uric acid level and coronary artery disease. Int J Clin Pract.

[12] Gur M, Yilmaz R, Demirbag R, Aksoy N (2008). Relation of serum uric acid levels with the presence and severity of angiographic coronary artery disease. Angiology.

[13] Duran M, Kalay N, Akpek M, Orscelik O, Elcik D, Ocak A (2012). High Levels of Serum Uric Acid predict severity of ischemic heart disease in Patients With Acute Coronary Syndrome. Angiology.

[14] Xiong Z, Zhu C, Qian X, Zhu J, Wu Z, Chen L (2011). Predictors of clinical SYNTAX score in coronary artery disease: serum uric acid, smoking, and Framingham risk stratification. J Invasive Cardiol.

[15] Lu P, Hu D, Lu J, Wang W, Chen B (2002). The association between uric acid and coronary heart disease. ZhonghuaNeiKeZaZhi.

[16] Puig JG, Martínez MA (2008). Hyperuricemia, gout and the metabolic syndrome. Curr Opin Rheumatol.

[17] Freedman DS, Williamson DF, Gunter EW, Byers T (1995). Relation of serum uric acid to mortality and ischemic heart disease. The NHANES I Epidemiologic Follow-up Study. Am J Epidemiol.

[18] Ndrepepa G, Braun S, King L, Fusaro M, Tada T, Cassese S (2013). Uric acid and prognosis in angiography-proven coronary artery disease. Eur J Clin Invest.

[19] Kojima S, Sakamoto T, Ishihara M, Kimura K, Miyazaki S, Yamagishi M (2005). Prognostic usefulness of serum uric acid after acute myocardial infarction (the Japanese Acute Coronary Syndrome Study). Am J Cardiol.

[20] Tamariz L, Agarwal S, Soliman EZ, Chamberlain AM, Prineas R, Folsom AR (2011). Association of serum uric acid with incident atrial fibrillation (from the Atherosclerosis Risk in Communities [ARIC] study). Am J Cardiol.

[21] Demir B, Caglar IM, Ugurlucan M, Ozde C, OktayTureli H, Ciftci S (2012). The Relationship Between Severity of Calcific Aortic Stenosis and Serum Uric Acid Levels. Angiology.

[22] Noman A, Ang DS, Ogston S (2010). Effect of high-dose allopurinol on exercise in patients with chronic stable angina: a randomised, placebo controlled crossover trial. Lancet.

[23] Ekelund UE, Harrison RW, Shokek O (1999). Intravenous allopurinol decreases myocardial oxygen consumption and increases mechanical efficiency in dogs with pacing-induced heart failure. Circ Res.

[24] Rekhraj S, Gandy SJ, Szwejkowski B, Nadir MA, Noman A, Houston JG (2013). High-Dose Allopurinol Reduces Left Ventricular Mass in Patients With Ischemic Heart Disease. J Am Coll Cardiol.

[25] Rajendra NS, Ireland S, George J, Belch JJF, Lang CC, Struthers AD (2011). Mechanistic insights into the therapeutic use of high-dose allopurinol in angina pectoris. J Am Coll Cardiol.

[26] Muir SW, Harrow C, Dawson J, Lees KR, Weir CJ, Sattar N (2008). Allopurinol use yields potentially beneficial effects on inflammatory indices in those with recent ischemic stroke: a randomized, double-blind, placebo-controlled trial. Stroke.

